# “How Will Medicaid Work Requirements Affect American Healthcare? A Look at What Past and Present Policy Tells Us”

**DOI:** 10.1177/00469580241251935

**Published:** 2024-05-24

**Authors:** Michael Folse, James Bridges, Anthony DiGiorgio

**Affiliations:** 1 LSU Health Shreveport School of Medicine, Shreveport, LA, USA; 2 Department of Neurological Surgery, University of California San Francisco, San Francisco, CA, USA; 3 Philip R Lee Institute for Health Policy Studies, University of California, San Francisco, CA, USA

**Keywords:** medicaid work requirements, SNAP, TANF, employment, income

## Abstract

Many social services have work requirements. Notably, Medicaid has no requirement that healthy, able-bodied beneficiaries work to receive benefits. There have been attempts at incorporating work requirement policies into several US states, but only a few have been implemented. The effect of work requirements has been studied in several other federally funded programs such as TANF, SNAP, and historically in the Civilian Corps created by Franklin Roosevelt. In general, these programs seem to have modest improvements in employment but are better when implemented with work supports which show improvement in employment and income. In this study, we examine the history of work requirements in Medicaid and other social programs to see which policies have the most effect on enrollment and employment.

**What do we already know about this topic?**
We know the legal status of Medicaid work requirements by state and their short-term effects. In addition, we know the effects of Medicaid and work support on employment and income.
**How does your research contribute to the field?**
Our research contributes to the field by describing the current state of Medicaid work requirements and their potential implications in 1 concise manuscript.
**What are your research’s implications toward theory, practice, or policy?**
Our research outlines the current state of Medicaid work requirements while also presenting the successes and failures in other programs with work requirements to show how a successful federal work requirement might be modeled.

## Introduction

Employment improves health,^1^ while unemployment is associated with poor health.^2^ Because of this, employment is considered one of the core social determinants of health (SDOH).^2^ The Biden Administration has made focusing on SDOH a priority in attempts at improving health outcomes in Medicaid.^3^ Given the strong correlation between employment and health, work requirements have been discussed by lawmakers as one way to promote that specific SDOH. This is not a novel discussion, as legislators have routinely tied social welfare programs to work requirements to encourage workforce participation. This goes back to the 1933 Civilian Conservation Corps started by President Roosevelt and includes work requirements that were enacted by Bill Clinton in the 1996 Personal Responsibility and Work Opportunity Reconciliation Act. Work requirements have also been tied to benefits in the Temporary Assistance for Needy Families (TANF) program, the Supplemental Nutrition Assistance Program (SNAP), and sporadically in Medicaid.

Politically, there is public support for work requirements in Medicaid,^4^ yet there is currently no federal requirement for them. Historically, those eligible for Medicaid were not expected to work, as Medicaid only covered the disabled, pregnant women, or children prior to the passage of the Affordable Care Act (ACA). The ACA allowed states to expand Medicaid to healthy, working-age adults, although not every state has expanded this coverage. Many expansion states, to promote SDON, have applied to the Medicaid section 1115 waiver program in attempts to institute work requirements. Additionally, non-expansion states have considered using work requirements to expand Medicaid to beneficiaries that would otherwise be covered by ACA Medicaid expansion. In all, 13 states had Medicaid work requirement waivers approved by CMS while another 9 had pending requests that had not been approved at the time of this writing (Tables 1 and 2).^5^ A summary of these waivers and requests is provided below (adopted from the Kaiser Family Foundation (KFF))^5^:

**Table 1.** States That Received Federal Approval for Work Requirements.

States	Requirement population	Hours required	Current status
Arizona	Expansion adults under age 50	80/month	Withdrawn June 2021
Arkansas	Expansion adults under age 50	80/month	Struck down by federal judge in 2019. New requirements being worked into the ARHOME project
Georgia^a^	Adults under federal poverty limit and under age 65	80/month	Implemented and allows expansion to otherwise ineligible populations
Indiana	Expansion and traditional adults under age 60	20/week	Waiver revoked
Kentucky	Expansion and traditional adults under age 65	80/month	Blocked by federal judge
Maine	Traditional adults under age 65	80/month	A waiver which included work requirements was approved by CMS in 2018, but the governor told CMS that the terms of the waiver would not be accepted by the state
Michigan	Expansion adults under age 63	80/month	Waiver revoked
Nebraska	Expansion adults under age 60	80/month	Waiver revoked
New Hampshire	Expansion adults under age 65	100/month	New Hampshire’s CMS waiver which was approved in 2018, was set aside by courts in 2019
Ohio	Expansion adults under age 50	80/month	Waiver revoked
South Carolina^a^	Traditional adults under age 65	80/month	South Carolina’s work requirement was split into two waivers: Healthy Connection Works waiver and the Palmetto Pathway waiver. Implemented and allows expansion to otherwise ineligible populations
Utah	Expansion adults under age 60	30/week	Waiver revoked
Wisconsin^a^	Traditional adults under age 50	80/month	Waiver revoked

a States that have not expanded Medicaid.

**Table 2.** States That Had a Pending Work Requirement Request but Never Received Federal Approval.

States	Requirement population	Hours required
Alabama^a^	Traditional adults under age 60	35/week (or 20/week for parents or caretakes with a child under the age of 6)
Idaho	Expansion adults under age 60	20/week
Kansas^a^	Traditional adults under age 65	20/week
Mississippi^a^	Traditional adults under age 65	20/week
Montana	Expansion adults under age 55	80/month
Oklahoma	Expansion adults under age 50	80/month
South Dakota	Traditional adults under age 60	80/month
Tennessee^a^	Traditional adults under age 65	20/week
Virginia	Expansion adults and traditional adults under age 65	80/month

a States that have not expanded Medicaid.

In this study, we review the status of the various Medicaid work requirement waiver requests and how work requirements have affected beneficiaries in other social benefit programs. We use these results to outline a framework for a Medicaid work requirement that improves the health of Medicaid beneficiaries.

## Examples of Medicaid Work Requirement Implementations

### Arkansas

Arkansas is the first and only state to successfully, and fully, implement a Medicaid work requirement.^6^ Taking effect in June 2018, Arkansas Medicaid beneficiaries between the ages of 30 and 49 were required to work 80 h per month, participate in alternate forms of community engagement (such as job training or community service), or qualify for an exemption (such as pregnancy or disability).^6,7^ Failure to comply or submit monthly reports for 3 months within a year led to the beneficiary having their Medicaid coverage revoked.^7^ After only 10 months of being implemented, the policy was put on hold as a federal judge ruled that the program did not satisfy the primary objective of Medicaid,^6^ being upheld on appeal. Arkansas has since incorporated a work requirement into its ARHOME program for Medicaid expansion under the ACA. This would require that able-bodied adults covered under the Medicaid expansion meet work requirements for qualified managed care plans. However, if they fail to meet those requirements, they are instead placed on Medicaid fee-for-service instead of losing all benefits entirely. The ARHOME program also includes “success coaches” to assist beneficiaries with job search and training.^8^

Over the 18 months that work requirements were implemented no significant changes in employment were seen. Of the target population it was estimated that 95% already met work requirements or should have qualified for an exemption. For the population that was not employed it was stated that job training or education and effective transportation to work would instead be state services provided that could benefit them most.^9^

The effects on coverage to beneficiaries following work requirements were significant. Sommers et al^9^ found that before the work requirement, Medicaid or marketplace coverage was 70.5% among Arkansans aged 30 to 49. After the implementation of the work requirement, the coverage fell to 63.7% but then increased to 66.1% when the work requirement was no longer in effect in 2019.^9^ The uninsured rate increased by 7.1% following the introduction of the policy but then returned to near normal following the court’s reversal.^9^

### Georgia

Georgia is the most recent state to implement work requirements for those receiving Medicaid, done so through their Georgia Pathways to Coverage program. Notably, Georgia has not expanded Medicaid coverage but rather stated that their goal of work requirements is to increase enrollment to a population that would be otherwise covered if the state had implemented Medicaid expansion.^10^

Initially approved during the Trump administration, these requirements were rescinded in early 2021. After being sued by Georgia Governor Brian Kemp, the Biden administration did not appeal, allowing the program to take effect on July 1st, 2023. Georgia’s Pathway to Coverage program allows beneficiaries to receive Medicaid coverage if they complete a minimum of 80 h of work, education, or community engagement each month to obtain and maintain Medicaid eligibility.^11^ To obtain benefits, applicants must demonstrate compliance during the month prior to application, and proof of compliance must be maintained for 6 consecutive months.^11^ If these 6 months are met, beneficiaries then only have to submit documentation annually.^11^ Georgia provides no exemptions for these work requirements, as it is intended to expand Medicaid benefits to individuals who would have otherwise been ineligible. Failure to make payments over 2 months results in a suspension of coverage (with termination of enrollment if payment is not made within 90 days).^11^ There is no data on the results of Georgia’s program thus far.

### California’s Working Disabled Program

California has implemented a program for certain working individuals with a disability to become eligible for Medi-Cal (California’s Medicaid program), known as the 250% Working Disabled Program (250% WDP). Although Medi-Cal itself does not have a work requirement, the WDP allows beneficiaries with higher incomes and assets to keep their Medi-Cal benefits. While California has expanded Medicaid coverage under the ACA, this work requirement is similar to the Georgia program in that it extends Medicaid coverage to those who would otherwise be ineligible. The 250% WDP requires that one must be working and have the ability to report payment to their local county welfare department.^12^ Unlike the requirements of Arkansas and Georgia, 250% WDP has no minimum hours or amount individuals must earn to be eligible.^12^ Other criteria to become eligible state that one must continuously meet the criteria of disability, as defined by the Social Security Administration (not engaging in any substantial gainful activity due to a medically determinable physical or mental disability for a minimum of 12 months),^13^ have a net income of less than 250% of the federal poverty level ($3038 per month for individuals, $4108 per month for couples), and be eligible for Supplemental Security Income/State Supplementary program benefits if not for one’s earned income.^12,14^ While participants were once required to pay monthly premiums based on their net income, a California law implemented in July of 2022 reduced premiums to 0$ while keeping Medi-Cal benefits the same.^12^

The early effects of these work requirements were investigated in a 2003 review by Jee and Menges^15^ of The Lewin Group. There does not seem to be concrete data on changes in employment, however, it is mentioned throughout the report that individuals did seek out employment simply to become eligible for the WDP. This was not without its challenges, however, as the disabled individuals for whom this program was created still faced barriers to both obtaining and maintaining employment. Once these individuals lose employment they lose eligibility for the WDP but can maintain Medi-Cal coverage by being reassigned to the Aged, Blind, and Disabled-Medically Needy aid category. This requires them to pay a share of their medical costs if their net countable income exceeds the limit for eligibility.^15^

### Utah

Utah was 1 of the 11 states to be granted a waiver in order to implement community engagement requirements as part of their full Medicaid expansion within the state.^16^ Beginning in January of 2020, Medicaid members must have completed the following activities within a 3-month window in order to maintain eligibility: create a job seeker account through Utah’s Department of Workforce Services, complete an online evaluation and set of online workshops, and apply for at least 48 jobs.^17,18^ This requirement would have to be met every year to maintain eligibility.^17^ The goal of this program was not to be cruel, however, as evidenced by the long list of exemptions from these community engagement requirements. These exemptions included: working at least 30 h each week (or working and earning the equivalent of 30 h a week at federal minimum wage), individuals age 60 to 64, pregnant individuals or individuals within the 60-day post-partum period, physically or mentally unable to meet the above requirements (as determined by a medical professional), responsible for the care of a dependent child under the age of 6 or the care of person with a disability recognized under federal law, individuals that are a member of a federally recognized tribe, individuals that are currently receiving unemployment insurance benefits (or awaiting an eligibility decision for these benefits), individuals participating in a regularly scheduled Substance Use Disorder (SUD) treatment program, individuals enrolled at least half time in any school, vocational training, or apprenticeship program, individuals participating in refugee employment services offered by the state, and individuals currently receiving SNAP and/or TANF benefits (or are exempt from their employment requirements).^17^ This community engagement requirement was in effect for only 3 months and was suspended on April 1st of 2020 as a result of the COVID-19 pandemic.^18^ These requirements were then fully withdrawn on August 10th of 2021 by CMS.^18^

The effects of Utah’s community engagement requirement are difficult to assess given its short-lasting time frame (only being implemented for 3 months) and correlation with the start of the COVID-19 pandemic. Little evidence exists on the policy’s direct effects on employment, as unemployment levels were already rising due to the ongoing public health crisis. This rise in unemployment, however, was one of the contributing factors in the decision to suspend these requirements, as noted by Utah’s Medicaid Director Nate Checketts.^19^

## What Challenges Have States Faced in Implementing Work Requirements?

Despite the intentions of policymakers, there remain significant challenges to work requirement implementation. These struggles are long-standing, as in 2002 the California Working Disabled program had only totaled 652 enrollees in its first 2 years of implementation, an amount much lower than the estimated 7000 to 14 000. A 2003 investigation into this poor enrollment noted poor program outreach, little knowledge about the program by eligibility workers, and general disinterest in the program’s benefits all to be contributing factors.^15^ More recent implementations of Medicaid work requirements show similar struggles. Sommers et al^9^ have reported on the work requirement implementation in Arkansas in their 2-year follow-up study post-implementation. Through the use of a random survey, their study highlighted the barriers to implementation that both policy administrators and beneficiaries faced. For most of 2018, the only method of reporting work and qualifying activities was through the use of an internet website on a computer, an option only 11.3% of Arkansans stated as their preferred method.^9^ More popular methods of reporting work and qualifying activities would have been through either a smartphone app/web page, or via calling a specified telephone number, which were the preferred methods for 32.6% and 27.8% of Arkansas residents, respectively.^9^ There also was decreased knowledge about the status of work requirements, as at the time of survey in late 2019, 70.8% of Arkansas residents reported that they were unsure if the policy was even still in effect.^9^

The effects of policies in other states cannot be studied as they are either too recent (Georgia) or were not successfully implemented (Utah).

## Effects of Work Requirements in Other Social Benefit Programs

Work requirements are not exclusive to Medicaid and various state and federal programs have implemented them in the past.

### FDR Civilian Corps

The FDR Civilian Conservation Corps (CCC) was established in 1933 as part of the New Deal. It was modeled as a work relief program for young men from unemployed families as a form of income supplement. Instead of simply sending checks to these families, it required that enrollees perform physical work in environmental conservation (planting trees, building parks, improving infrastructure, etc.). The participants were paid but most of the funds went back to their families. With over 2.5 million participants it was widely successful and provided skills that could be transferred to other employment after leaving the CCC. Approximately $663 million was sent to the dependent families of the beneficiaries over the 9 years of the program.

### Supplemental Nutrition Assistance Program

To participate in the Supplemental Nutrition Assistance Program (SNAP), 2 work requirements must be satisfied. First, unless the individual already works over 30 h a week or is taking care of children under 6 years old, they must accept a job if offered one.^6^ Secondly, if they are able-bodied and under age 50 without any dependent children, their benefits will be limited to 3 months over a 3 year period.^6^ The exception to this is if the participant is in job training or works more than 80 h each month.^6^ In able-bodied adults without dependents (ABAWDS), which only constituted about 7% of SNAP recipients in 2019,^20^ employment was found to slightly increase.^6,21^ Other studies have found that work requirements decrease participation in SNAP by as much as 53%, but found no effects on employment.^22^

### Temporary Assistance for Needy Families

The Temporary Assistance for Needy Families (TANF) gives states the flexibility to assist families with children who are in need through monthly cash payments.^23^ The overarching goal of this program is to ensure children are adequately cared for, to promote jobs and marriage, to reduce out-of-wedlock pregnancies, and to support families staying together.^23^ Starting in the 1990s, work requirements stated that at least 50% of parents receiving payments from TANF had to either work a certain number of hours or spend a certain amount of time in activities that could help them attain employment, such as education.^6^ After the implementation of work requirements for TANF, employment significantly increased for single mothers, who constitute the largest recipient of TANF funds.^6^ The income of employed single mothers has also increased, possibly due to the increased speed at which single mothers found a job, thus spending less time in the TANF program.^6^ Despite this increase, the effect of work requirements on all single mothers has been small, which may be attributed to the fact that around half of single mothers who leave TANF are not employed when they stop receiving TANF assistance.^6^

## Effects of Work Supports on Employment and Income

The effects of work requirements cannot be examined in isolation, as many have come with support programs to assist beneficiaries in finding and maintaining employment. This is largely modeled after the CCC, which improved the long-term employability of its participants. When paired with employment assistance programs, such as in TANF, work requirements are successful in increasing both the employment and earnings of beneficiaries.^24^ As the federal TANF program provides grants to states, and the program has work requirements, states are responsible for providing employment assistance to ensure it continues to receive funding.

## Modeling a Federal Medicaid Work Requirement

A federal Medicaid work requirement, if properly implemented, could achieve the stated goals of improving health. By addressing unemployment, a core social determinant of health, work requirements would improve the well-being of Medicaid beneficiaries. Poor employment has been associated with worsening physical and mental health as well as signs of decreased social and workplace well-being.^25-29^ A higher extent of poor employment has been associated with decreases in general physical health^26^ as well as more days of poor physical health and more days with limitations on activity due to health problems.^27^ Highly precarious work has been associated with negative emotions and lower life satisfaction while secure meaningful work was found to be associated with more positive emotions and greater life satisfaction.^28^ The link between work and health is strong and Medicaid should address that SDOH.

The work requirement must only be applied to able-bodied adults without dependents, as was the case with the SNAP benefits. The requirements would not apply to the elderly, disabled, pregnant women, or children, only to Medicaid beneficiaries who receive coverage under the Affordable Care Act expansion. While this is a low percentage of total Medicaid recipients, they also receive much more funding from Federal funds. ACA expansion beneficiaries have 90% of their funds covered via the Federal Medical Assistance Percentage (FMAP), much more than non-ACA expansion beneficiaries (50%-77% depending on the state).

The states can ensure their beneficiaries don’t lose coverage by creating work support programs. Federal job support programs are largely ineffective and thus empowering states to create job training programs is likely to lead to better outcomes.^30^ States can additionally apply for federal grants to assist in creating work support programs. As these programs will ultimately increase beneficiary health and keep their expenses heavily subsidized by FMAP, they can be designed in a revenue-neutral manner for the states. Additionally, this will likely allow states that have yet to expand their Medicaid eligibility to do so in both a financially and politically responsible manner.

The Georgia example is worth noting again here. Georgia is spending more on its Pathways to Coverage enrollees than it would if it simply expanded Medicaid under the ACA. ACA Medicaid expansion has 90% of beneficiary funding from federal sources, whereas Georgia is responsible for 100% of funding beneficiaries added to Medicaid under Pathways to Coverage. Georgia finds this preferable to ACA expansion and a 1115 Medicaid work requirement waiver, as experience from other states shows those waivers can be rescinded with a new chief executive. A federal work requirement would allow Georgia (and other expansion holdouts) to expand Medicaid coverage while allowing it leeway to use employment as a means to improve health.

## Conclusion

The use of Medicaid work requirements in the United States has been extremely limited. It is difficult to determine the effects that these policies have had on factors such as employment, income, and program enrollment because they were in effect for a short period or there has not been time since their implementation to make strong conclusions. A look at other federally subsidized programs that have work requirements such as SNAP and TANF generally appear to improve employment. For work requirements to be effective, the addition of work support programs including job search assistance, job training, and subsidized child care is crucial. The success of the CCC shows what this could look like on a federal level. Careful consideration must be taken for these factors if legislation is to be created for a federal Medicaid work requirement.
